# The influence of prime characteristics in semantic priming

**DOI:** 10.3389/fpsyg.2026.1680365

**Published:** 2026-03-04

**Authors:** Ajay Mangat, Alexander Taikh, Christina Gagne, Thomas L Spalding

**Affiliations:** 1 Department of Psychology, Concordia University of Edmonton, Edmonton, AB, Canada; 2 Department of Psychology, University of Alberta Faculty of Arts, Edmonton, AB, Canada

**Keywords:** automatic spreading activation, lexical decision task (LDT), semantic categorization task, semantic priming, strategic processing, typing task

## Abstract

The semantic priming effect refers to the finding that processing a target word is facilitated when it is preceded by a related prime compared to an unrelated prime, and the semantic priming paradigm has been a key way to examine how knowledge is organized and retrieved. Both automatic and strategic processing of the prime can contribute to the semantic priming effect, and a major challenge in developing an integrated account of semantic priming is delineating conditions where the effect is driven by automatic or strategic processing of the prime. We incorporate literature reviews, meta-analyses, and studies with original experiments to examine how numerous factors influence the automatic and strategic processing of the prime, and thus the semantic priming effect. Specifically, we discuss the foundational models of semantic priming, and the automatic and strategic mechanisms by which primes are thought to influence their targets. We then discuss how prime-level lexical and semantic properties, prime-target relational factors, modality and presentation variables, and task-level determinants influence the automatic and strategic processing of the prime. We also point out gaps in the literature and suggest next steps in developing an integrated account of semantic priming.

## Introduction

The semantic priming effect (Meyer and Schvaneveldt, 1971) is the finding that processing a target word is facilitated when it is preceded by a prime that is semantically related to its target (e.g., processing of the target word *dog* is facilitated by the related prime *cat* compared to an unrelated prime *table*). The semantic priming paradigm has been used to examine the organization and retrieval of semantic knowledge, and how contextual semantic information influences the word recognition process (e.g., see Jones and Estes, 2012; Neely, 1991 for reviews). Both automatic and strategic processing of the prime can contribute to processing of the target and the semantic priming effect (Neely et al., 1989; Perea and Rosa, 2002). Automatic processing of the prime occurs quickly, and does not require attention or the awareness of the prime (Posner and Snyder, 1975), while strategic processing of the prime requires its conscious identification, takes more time, and is more likely to be influenced by various experimental factors, such as the linguistic properties of the prime and its relationship to the target, how the prime is presented, and the type of task being used. Importantly, strategic processing of the prime can mask the contribution of automatic prime processing to the semantic priming effect.

Studies that use the semantic priming paradigm to examine the nature of semantic representations have generally used factorial designs and have examined the influence of the type of relationship that exists between primes and targets on the semantic priming effect (e.g., Bueno and Frenck-Mestre, 2008; Lucas, 2000; Perea and Rosa, 2002) and the effect of the experimental task and list context effects on the semantic priming effect (e.g., Becker et al., 1997; Bueno and Frenck-Mestre, 2008; de Groot, 1990; de Wit and Kinoshita, 2014, 2015a, 2015b; Grainger, 1998; Perea and Rosa, 2002; Williams, 1996). While the linguistic properties of primes and targets influence their processing, studies have generally focused on the influence of linguistic variables on the recognition of individual words outside of the priming paradigm (e.g., Pexman et al., 2008; Yap et al., 2012; Yap et al., 2011), or have focused on the linguistic properties of the target word (e.g., Becker, 1979; Cortese et al., 1997; Hutchison et al., 2008). However, less direct emphasis has been placed on the processing of the prime itself, which is critical to understanding the semantic priming effect, and distinguishing between the influences of automatic and strategic processing.

The central aim of this review is to examine how various factors that influence the processing of the prime modulate the balance between automatic and strategic processing. Delineating the conditions under which automatic and strategic processing of the prime drive or contribute to the semantic priming effect is critical to developing an integrated account of semantic priming. We draw upon meta-analyses, literature reviews, and studies reporting original experiments. We first provide an overview of the foundational models of semantic priming, which are distinguished by whether the influence of the prime is automatic or strategic, and by whether the prime facilitates the identification of the target or whether the two are evaluated together after identification of the target. We then focus on factors that influence the processing of the prime: the prime-level lexical and semantic properties; prime-target relational factors; modality and presentation variables; and task-level determinants. We then describe an integrated account of semantic priming (Perea and Rosa, 2002) which posits both automatic and strategic processing of the prime, and discuss the gaps in the current literature and the challenges to developing an integrated account of semantic priming. Our aim is not to provide a fully unified model of the semantic priming effect, but to delineate boundary conditions between when semantic priming is driven by automatic or strategic processing and to highlight the joint influence of numerous factors (prime-level properties, prime-target relational factors, modality and presentation variables, and task-level determinants) on the semantic priming effect whenever possible.

## Foundational models of semantic priming

A common conception is that a lexical network (i.e., the mental lexicon) stores words based on their form and sound, whereas, the semantic information associated with words is thought to be stored in a separate network. Indeed, there are numerous models (e.g., Anderson, 1983; Collins and Loftus, 1975; Seidenberg and McClelland, 1989; Plaut, 1995; Plaut and Booth, 2000) that make this distinction when describing how words and their associated meanings are stored and retrieved. For example, according to localist models (e.g., Collins and Loftus, 1975), the concepts associated with words are stored as units in a semantic network and are connected based on associations that are learned over time (e.g., *cat* and *dog*). Activation of one conceptual node (i.e., *cat*) results in activation of the connected nodes (i.e., *dog*). According to distributed models (e.g., Plaut, 1995), a semantic network is composed of features, and a concept corresponds to a set of related features and other concepts (e.g., the features of fur, a tail, four legs… are associated with both the concept of cat and the concept of dog).

In experiments examining the semantic priming effect, the prime target pairs can be related associatively, where the occurrence of one word is predictive of the occurrence of the other, and/or semantically, where the prime and target overlap in features or have any kind of a meaning relationship (e.g., synonyms, antonyms…). Localist models thus generally explain semantic priming as activation automatically spreading from the node that represents the prime to related nodes and thus facilitating processing of those words (e.g., the node of the target word, *dog*, receives activation from the node of the prime word, *cat*, facilitating the processing of the target word). Distributed models, in contrast, assume that the features activated by the prime will help facilitate the processing of the target to the extent that the prime and target have overlapping features.

While numerous mechanisms have been proposed to explain the semantic priming effect, they generally involve two key distinctions. The first distinction is whether processing the semantic prime influences the lexical activation of the target word or whether the prime and the target are processed together after the lexical selection of the target word. The second distinction is whether the process responsible for the priming effect is automatic or strategic. In this section, we briefly review three types of accounts of semantic priming that highlight these distinctions.

### Automatic processing of the prime

Automatic spreading activation, as first explained by Collins and Loftus (1975; see also Posner and Snyder, 1975), proposes that the activation from the lexical representation of the prime spreads to the target’s lexical representation. Thus, the prime pre-activates the target’s lexical representation and influences the lexical selection of the target. According to localist accounts of semantic memory, such as the one proposed by Collins and Loftus, the facilitation of the target occurs through direct links or connections within semantic memory because the prime and target are associated with each other. Thus, there is no concurrent processing of the prime and target. Spreading activation occurs quickly and automatically (Posner and Snyder, 1975) in that it does not require attention, or even explicit awareness of the prime. We should also note that distributed accounts of semantic memory (Fernandino and Conant, 2024; Fischler, 1977; Plaut, 1995) also potentially explain fast acting and automatic semantic priming effects. For example, de Wit and Kinoshita (2014, 2015a, 2015b) propose that the features of the prime are extracted and facilitate the processing of the target, although this process depends on participants having a task that relies on encoding features (we discuss these studies and the effect of task type, subsequently). The processes of expectancy generation and retrospective post-lexical integration (see Neely, 1991 for a review) which we describe next, can contribute to the semantic priming effect concurrently, and thus obscure the influence of automatic spreading activation.

### Expectancy generation

According to expectancy accounts of semantic priming (Becker, 1980; Neely, 1977), participants predict which target words are likely to follow the prime, or the relevant features that define the target words. An expected set of words (or features) should facilitate the recognition of a target related to the prime, but also delay responding to an unrelated target, as participants would first have to reject the words in their expectancy set and then consider the unrelated target word. Expectancy can be a strategic process which requires attentional resources, and the formation of expectancy sets is thought to require enough time between the onset of the prime and the onset of the target (i.e., stimulus onset asynchrony, SOA), and is encouraged by having a greater proportion of primes and targets be related to each other (i.e., relatedness proportion). The SOA needed for expectancy generation, or other strategic processes, to occur is unclear and likely varies between tasks (Hutchison et al., 2001), but has been estimated to be about 300 ms (Neely, 1991).

### Post-lexical integration

Retrospective post-lexical and meaning integration accounts posit that participants process the prime and target together. The prime does not influence the lexical selection of the target. Instead, participants are thought to engage in a semantic matching process where they determine whether the prime and target are semantically related following lexical access of the target word but prior to the response of the experimental task (de Groot, 1984; de Wit and Kinoshita, 2014, 2015a, 2015b; Forster, 1981; McKoon and Ratcliff, 1992; Neely et al., 1989). For example, in the compound cue model (McKoon and Ratcliff, 1992) participants form a compound cue made up of the prime and target, and evaluate its familiarity. Familiar cues are ones where prime and target are associatively or semantically related. Similar to expectancy accounts, this process is under strategic control, at least to some degree, and requires processing the meaning of the prime (Neely and Keefe, 1989), which can occur before or after the presentation of the target. In the lexical decision task (LDT), where participants judge whether the target letter string is a word or not, retrospective integration accounts explain the semantic priming effect as a bias. Specifically, participants are biased to make a “word” response when the prime and target are related. The detection of a relationship between the prime and target, such as a familiar prime-target compound cue, would require that the target be a word. Thus, participants are biased to make a “nonword” response when prime and target are unrelated, and are slowed in their response to words following unrelated primes. Increasing the relatedness proportion thus also encourages the use of retrospective integration to guide the LDT decision, because “word” responses are more likely to come after related primes. Thus, retrospective matching can be potentially confounded with expectancy generation.

Importantly, multiple mechanisms can potentially contribute to the semantic priming effect because the processing of the prime can continue when it is no longer being shown and can overlap with the processing of the target. For example, when the duration of the prime and the SOA are sufficient (i.e., over 300 ms), the priming effect can potentially be explained by expectancy generation, given that there is enough time to develop expectancy set, by retrospective matching, since the processing of the prime can continue after the presentation of the target, and potentially, automatic spreading activation since its influence may persist throughout the trial. Indeed, Perea and Rosa (2002) propose a hybrid model of semantic priming, which includes spreading activation and post-lexical integration accounts (see also Neely et al., 1989). We discuss Perea and Rosa in more detail subsequently, but briefly, the authors propose that automatic spreading activation drives semantic priming when the prime is briefly presented and the onset of the target follows quickly enough so that automatic spreading activation does not fade. Post-lexical integration would then happen after accessing the target word.

As the brief overview of accounts of semantic priming demonstrates, the influence of automatic prime processing on the semantic priming effect is difficult to rule out, but may be obscured by the strategic processing of the prime. Strategic prime processing is more flexible and more likely to be influenced by numerous factors that can be divided into prime-level lexical and semantic properties, prime-target relational factors, modality and presentation variables, and task-level determinants (though these variables can potentially influence the automatic processing of the prime as well). We first focus on prime-level lexical and semantic properties, which influence identifying the prime word and extracting its semantic information that drives the semantic priming effect.

## Prime-level lexical and semantic properties

Most studies on word recognition have examined the influence of the linguistic item-level properties of individual words, outside of the semantic priming paradigm. Although these studies do not directly use a semantic priming paradigm, how a word’s lexical and semantic information influences its own recognition may provide insight into how that word would be processed as a prime and thus influence the processing of a subsequently presented target. For example, properties that facilitate the recognition of the word and access to its semantic information might make it a more effective semantic prime.

### Lexical properties

The influence of lexical factors on word recognition has been well studied (see Yap and Balota, 2015 for a review) and a review of this topic is beyond the scope of the current paper. However, some key lexical factors are frequency (e.g., Whaley, 1978), where words that occur more frequently in the language are recognized more quickly, and length, where shorter words are recognized more quickly (although length and frequency are strongly inversely correlated). Words with a greater orthographic neighborhood size (i.e., the number of words that can be obtained by substituting a single letter of the target word) are responded to more quickly in naming and the lexical decision task, especially for low frequency words (see Andrews, 1997 for a review). These lexical properties influence how quickly the lexical representation of a word is accessed, and thus how quickly the semantic information of a word is accessed. Indeed, Taikh et al. (2015) found that access to meaning in words first requires lexical access, which depends on lexical variables. Taikh et al. examined the influence of lexical and semantic variables for words and their corresponding images on a semantic classification task (living vs. non-living). Lexical variables did not predict response latencies for images, but they did predict response latencies for words.

Less is known about the influence of linguistic properties of primes and targets on the semantic priming effect. Becker (1979) showed that semantic priming effects were larger for lower frequency targets. Hutchison et al. (2008) collected lexical decision and naming responses to 300 semantic prime-target pairs from over 200 participants. This early semantic priming megastudy was extended by the Semantic Priming Project (Hutchison et al., 2001), which we will discuss in more detail subsequently. Using a large set of items and participants allowed Hutchison et al. (2008) to examine the influence of both prime and target item-level variables on the semantic priming effect. In their experimental paradigm, the authors used primes that were semantically related to their targets, semantically unrelated, or were neutral primes (i.e., the prime was the word “BLANK”). The SOA was either short (250 ms) or long (1,250 ms). The responses to targets following neutral primes were used as an indication of how difficult the target was to recognize in isolation. Priming was greater for targets that had longer latencies in the neutral priming condition. However, in the LDT, priming was greater for shorter targets and targets with fewer orthographic neighbors when the SOA was long, which is somewhat contradictory to their finding of greater priming effects for words that were responded to more slowly in the neutral priming condition, and to findings that greater orthographic neighborhoods facilitate the recognition of individual words (Andrews, 1997). In general, however, Hutchison et al. argue that a related prime may have a greater impact on the target when the target is processed more slowly, allowing more time for the related (vs. unrelated) prime to influence the recognition of the target.

In terms of the characteristics of the prime, Hutchison et al. (2008) found that in both the lexical decision task and naming, priming was greater when related primes were short and had fewer orthographic neighbors. These results are stronger at short SOA’s, where priming was also greater for high-frequency related primes (we discuss the influence of SOA on the priming effect subsequently). The authors argue that prime words that are recognized and identified more quickly can facilitate the recognition of their targets to a greater extent, especially at short SOA when the influence of strategic processes may be reduced. Interestingly, and in contrast to these findings, Hutchison et al. also found that priming in the LDT, but not in naming, was greater for prime words that were responded to more slowly in the ELP database, and this effect was greater at long SOA. The authors did not offer an explanation for this contradictory finding.

### Semantic properties

Similarly, words with more semantic information associated with them are processed faster, especially in tasks that require semantic information about the word (see Balota et al., 1991; Pexman, 2012, 2020 for reviews). The retrieval of meaning is facilitated when the word has a richer semantic representation (e.g., Barca et al., 2016; Pexman et al., 2008; Sidhu et al., 2016; Taikh et al., 2015; Yap et al., 2011). For example, responses to words are facilitated when the word has a greater semantic neighborhood size (i.e., the number of semantically related words or concepts that a word has) (Buchanan et al., 2001), when the word has more semantic features associated with it (McRae et al., 2005), and when it elicits more first associates in a free association task (Nelson et al., 1998).

Studies have also examined semantic priming where the primes are novel words learned by the participants during the experiment (e.g., Bakker et al., 2015; Bakker-Marshall et al., 2018; Balass et al., 2010; Ball et al., 2024; Borovsky et al., 2012; Perfetti et al., 2005; Tamminen and Gaskell, 2013), with mixed results on whether newly learned words can be effective semantic primes. Novel words, even if adequately learned in the context of the experiment, seem to be processed differently as primes from familiar words. In general, novel words semantically primed their targets when they were able to be processed strategically because of a long enough SOA and a task that encourages strategic processing. We discuss the influence of both of these factors on novel word semantic primes subsequently. In general, however, semantic information associated with novel words is likely not integrated in the semantic network and thus not connected with semantic representations of familiar words (Davis and Gaskell, 2009), meaning that these words, as primes, could not automatically influence their targets. Semantic information associated with novel words should be activated more slowly and they are only able to act as semantic primes if they can be processed strategically.

In summary, the literature suggests that, in general, quicker processing of the prime predicts a greater semantic priming effect, especially at shorter SOAs where automatic processing of the prime is thought to play a greater role. At longer SOAs, the linguistic properties of the prime and strategic processes may override the relatively subtle facilitation provided by the prime being easy to process. Slower processing of the targets likewise predicts a greater priming effect because it allows the related prime to exert its influence. However, the way semantic information from the prime and from the target are processed and influence responding to the target depends on the prime-target relationship. Specifically, information extracted from the prime is most useful if it has a strong associative relationship to the target and can prospectively facilitate its processing. We focus on the influence of prime-target relationships next, which help determine what information from the prime becomes relevant in processing the target and influencing the semantic priming effect.

## Prime-target relational factors

As noted previously, the relationship between a prime and target can be associative (Thompson-Schill et al., 1998), where the occurrence of one word is predictive of the occurrence of the other, and/or the relationship can be semantic, where the prime and target overlap in features or have any kind of a meaning relationship (e.g., synonyms, antonyms, and so on). The direction and strength of association can also influence the semantic priming effect. For example, priming in prime-target pairs with a strong forward association (e.g., cigar-smoke) may be driven by a different mechanism than prime-target pairs with a backward association (e.g., herd-cow).

### Associative and semantic prime-target relationships

Studies have attempted to determine whether automatic semantic priming is driven by the associative relationship between the prime and target, which is consistent with localist theories of how knowledge is stored, or by featural overlap, which is consistent with distributed theories of how knowledge is stored. One way that studies have attempted to distinguish between these drivers of semantic priming is by examining the effect of the type of relationship between prime and target on the semantic priming effect. Examining the effect of relationship type on semantic priming provides information on how primes are processed. For example, finding that featural overlap drives semantic priming would suggest that while processing the prime, its features are extracted and used to help process the target.

In a meta-analysis, Lucas (2000) analyzed priming effects from studies that apparently controlled the association strength in their prime-target pairs. Lucas found priming effects when the prime and target were related semantically in the absence of any associative relationship, but no priming based on just association (i.e., when there was no feature overlap). However, the effect was also stronger when the prime and target had both a semantic and an associative relationship. Priming effects driven purely by a semantic relationship were not influenced by SOA and relatedness proportion, suggesting they were driven by an automatic pre-activation mechanism rather than a strategic mechanism. One such effect was between category coordinates (i.e., where the prime and target belong to the same category) that are not associated with one another. Lucas concluded that semantic priming effects, at least those that are automatic, are driven primarily by feature overlap rather than semantic association. Similarly, Perea and Rosa (2002, Experiment 2) found priming in pairs that were semantically related (prime and target were synonyms, antonyms, or category coordinates) but not associated with each other according to free-association norms. We discuss Perea and Rosa in more depth subsequently.

Hutchison (2003) examined the studies used by Lucas (2000) and likewise concluded that priming for semantically related pairs was likely to be automatic, and that the priming effect was larger when prime-target pairs had both a semantic and associative relationship. However, in contrast to Lucas (2000), Hutchison concluded that there was strong evidence for semantic priming effects based on just association between prime and target. For example, Hutchison argued that in studies that Lucas used as evidence of priming between pairs that are semantically but not associatively related (e.g., category coordinates), the prime and target pairs actually were associatively related. Hutchison also argues that Perea and Rosa (2002) defined their prime target pairs as not associated based on the absence of only forward association strength, where the target is likely to be a response when the prime is a cue. The association between primes and targets can also be backward and symmetrical (we cover this subsequently). In general, Hutchison argued that isolating the influences of association and feature overlap is often difficult because words that are associated often also overlap on features, making it difficult to determine whether an observed priming effect is driven by an associative relationship or the feature overlap between primes and targets.

Hutchison (2003) further supports the presence of automatic associative priming (i.e., priming due to spreading activation) with mediated priming studies, which Lucas (2000) did not include in her meta-analysis. In the mediated priming paradigm, a target is related to a prime via a connecting word or concept (e.g., the target *stripes* is indirectly related to the prime *lion* through the mediating concept of *tiger*). Mediated priming has been found in the LDT (e.g., McNamara and Altarriba, 1988; McKoon and Ratcliff, 1992; Livesay and Burgess, 1998) and naming (e.g., Balota and Lorch, 1986; Livesay and Burgess, 1998), although see de Groot (1983) for evidence against mediated priming. Hutchison argues that mediated priming effects are likely automatic because strategic processing of the prime, while useful for direct associates, would not be useful for mediated associates. Specifically, using the retrospective matching strategy on mediated associates should bias participants to make nonword responses because the compound cue formed by the prime and target are not directly related, similarly to what would happen in a trial when the prime is unrelated to the target. A similar logic would apply to expectancy generation. Hutchison likewise argues that mediated priming is likely not due to feature overlap because the prime and target share little or no overlap in semantic features, although we are unaware of any studies directly examining the influence of feature overlap on mediated priming.

Other researchers argue that mediated priming could be explained by post-lexical matching strategies. For example, McKoon and Ratcliff (1992), argue that mediated priming effects may be due lexical co-occurrence, where the prime and target may not be associatively related, but may nonetheless co-occur in natural language. The familiarity of the prime-target compound cue would thus facilitate the processing of the target. However, Hutchison (2003) points to studies where the degree of association did not influence the mediated priming effect (Balota and Lorch, 1986; McNamara, 1992), and where lexical co-occurrence did not influence the mediated priming effect (Livesay and Burgess, 1998). Hutchison also mentions the findings of Hayes and Bissett (1998), where participants formed associations between novel words (i.e., nonwords). Specifically, participants were trained to associate stimuli from list A to corresponding stimuli from list B, and to corresponding stimuli from list C. Using a double LDT, where two letter strings are simultaneously presented and participants decide whether both are words or nonwords, Hayes and Bissett found that participants responded more quickly to pairs from lists B and C that were associated to each other though list A stimuli (although list B stimuli were never directly associated with list C stimuli). The findings of Hayes and Bissett are consistent with automatic spreading activation facilitating concepts that are linked in the semantic network, even if they do not co-occur.

More recently, Jones (2010) found evidence for a strategic post-lexical integration account of mediated priming. Jones used prime target pairs where there was no associative relationship between the prime and the mediator, and the mediator and the target. Jones found a mediated priming effect in the double and standard LDT tasks, but not the continuous LDT task, which precludes strategic processing. We discuss the continuous LDT in more detail subsequently. However, Jones argues that the search for a relationship between prime and target, as posited by semantic matching models (e.g., Neely et al., 1989), could include searching for a mediator (i.e., searching for *tiger* when shown the prime *lion* and the target *stripes*). Jones concludes that mediated priming is retrospective and strategic, although not in a way that is consistent with the compound-cue model (Ratcliff and McKoon, 1988).

### Direction and strength of association

Associative strength can be measured as the likelihood that the target word is produced as a response to the prime cue in a free association task (De Deyne et al., 2019; Nelson et al., 1998), and it can be directional. Specifically, the forward associative strength (FAS) is the proportion of participants who responded with the target when cued with the prime, while the backward associative strength (BAS) is the proportion of participants who produce the prime when cued with the target. According to the model of semantic priming proposed by Neely and Keefe (1989), stronger FAS should result in greater priming at longer SOAs, where participants would have more time to generate a list of expected targets given the prime. Stronger BAS was likewise predicted to increase the semantic priming effect at longer SOAs because participants are more likely to evaluate the target and the prime together using retrospective matching at longer SOAs. Hutchison et al. (2008) found that at a short SOA, greater FAS predicted a greater semantic priming effect. At long SOA, greater semantic priming was predicted by both stronger FAS and BAS. We will discuss these findings in more detail when considering task type. However, these findings are consistent with the idea of both retrospective matching and expectancy generation being applied at long SOA, but retrospective matching being less likely at a short SOA. One possibility for why a shorter SOA would reduce retrospective matching is that it does not allow enough time to process the meaning of the prime, as argued by Neely and Keefe (1989). However, the meaning of the prime may also be processed while the target is being processed.

The direction of association between prime and target can also influence the nature of the semantic priming effect. For example, Thomas et al. (2012) tested the semantic priming effect with pairs that only had a strong forward association (e.g., cigar—smoke), only a strong backward association (e.g., herd - cow), or were symmetrical (e.g., girl—boy). The targets were visually degraded. A common finding is that the semantic priming effect is greater for visually degraded targets (e.g., Balota et al., 2008; Borowsky and Besner, 1993; Stolz and Neely, 1995) because the longer time needed to process the target allows for more influence from the (related) semantic prime. Thomas et al. found a stronger semantic priming effect for visually degraded targets only when the prime and target pairs were backward associated or symmetrical. The authors argue that retrospective use of the prime is encouraged by the visually degraded target when there is a backward association.

Associative strength can also be measured in terms of the co-occurrence of the prime and target words, which is how likely they are to appear together in text. The latent semantic analysis (LSA) model (Landauer et al., 1998) assumes that semantically similar words co-occur within the same contexts. LSA values have been found to predict semantic priming effects (e.g., Chwilla and Kolk, 2002; Landauer and Dumais, 1997; Lund et al., 1995, 1996). Hutchison et al. (2008), however, found that LSA did not predict semantic priming effects. More recently, Fernandino and Conant (2024) compared how well multiple types of models predicted the automatic semantic priming effect in the LDT. In their experiment, primes were presented for 150 ms and were followed by a 50 ms mask, prior to the presentation of the target, thus precluding expectancy generation, although retrospective matching would still be possible since the primes were visually identified. Fernandino and Conant found that experiential similarity between primes and targets (i.e., sensory-motor and affective features of the words) was the primary driver of the semantic priming effect. Additionally, the authors did not find independent contribution of the co-occurrence measures or of taxonomic information (i.e., semantic similarity between prime and target based on their category information). The authors argue that sensori-motor and affective features of primes and targets are automatically activated during the LDT, which does not explicitly require that kind of information.

In summary, priming that is driven more by automatic processing of the prime seems to be more robust for semantically related pairs, however there is disagreement about whether automatic priming exists for pairs that are purely associatively related. Strong FAS produces robust priming at both long and short SOAs, consistent with a priming mechanism that is automatic and prospective (e.g., spreading activation). Priming driven by a strong BAS seems robust at long (but not short) SOAs, suggesting that BAS encourages the strategic and retrospective processing of the prime. However, the influence of the prime-target relationship on the priming effect depends on modality and presentation variables. For example, Perea and Rosa (2002) found that the effect of prime-target strength of association depends on SOA. We discuss Perea and Rosa in more detail subsequently.

## Modality and presentation variables

We first address the question of whether semantic priming occurs when the prime duration is too brief for it to be identified. A semantic priming effect without prime identification is consistent with the automatic accounts of semantic priming, such as spreading activation from the prime (Marcel, 1983), or accumulation of features from the prime (de Wit and Kinoshita, 2014, 2015a, 2015b), because strategic use of the prime (either via expectancy generation or retrospective matching) would be ruled out. We then examine studies where the prime can be consciously identified, and thus where its strategic processing is possible.

### Prime duration

Numerous studies have found semantic priming without prime identification (e.g., Carr et al., 1982; Cheesman and Merikle, 1986; Hirshman and Durante, 1992), while other studies found no semantic priming when the prime was not identified (Dark, 1988; Dark and Benson, 1991; de Wit and Kinoshita, 2015b). Specifically, Hirshman and Durante presented participants with prime - target pairs and asked participants to only identify the prime on some trials, and to only respond to the target in a LDT on others. The authors argue that measuring prime identification when participants are asked to also respond to targets can alter prime identification because of retrospective priming mechanisms (Dark and Benson, 1991), where information from the target helps participants identify a briefly presented prime. Masked primes that were presented for 33 ms were almost never identified, although a semantic priming effect was found. A semantic priming effect was also found when masked primes were presented for 50 ms, and these primes were more likely to be identified (about 50% when the prime was related to the target and about 30% when the prime was unrelated). The authors conclude that semantic priming can occur without identifying the prime (i.e., at 33 ms durations), although identification of primes can occur even when they are presented briefly and masked (i.e., at 50 ms durations). Importantly, even brief prime duration and a short SOA allows for the possibility of retrospective matching, since primes were more likely to be identified in prime-target pairs where a semantic priming effect was found, and when the prime and target were related. The semantic priming effect found by Hirshman and Durante when the prime was not identified is consistent with automatic spreading activation contributing to the semantic priming effect. However, post-lexical mechanisms may contribute to the effect when the prime is identified, even when SOA is short enough to not allow for the generation of expectancy sets.

Studies where primes are visible (where the prime duration is typically 100 ms or more) often manipulate the relatedness proportion, which is the proportion of related prime-target pairs in the experimental stimulus list (Tweedy et al., 1977), often in concert with manipulating the SOA. A semantic priming effect that is driven by automatic spreading activation, as conceptualized by Collins and Loftus (1975) should not be influenced by relatedness proportion. However, relatedness proportion should influence expectancy generation and retrospective matching. Participants are more likely to generate an expectancy set of the target, and evaluate the target and prime together, when they see the target when the relatedness proportion is high (Becker, 1980; Neely, 1977). Consistent with this notion, numerous studies (Grossi, 2006; Hutchison et al., 2001; Neely et al., 1989; Pecher et al., 2002; Perea and Rosa, 2002) have found that greater relatedness proportion increases the semantic priming effect at longer SOAs (see Hutchison, 2007) for a review. Effects found at short SOAs (under 300 ms) are thus thought to reflect automatic spreading activation (Neely, 1977), overlap of features between prime and target (de Wit and Kinoshita, 2014, 2015a, 2015b), and/or retrospective matching (e.g., Taikh and Lupker, 2020), but not expectancy generation. Of note, priming effects found at longer SOAs can be potentially explained by expectancy generation, retrospective matching, and automatic spreading activation, since it is unclear how long the influence from spreading activation can persist. However, retrospective matching requires enough time to process the meaning of the prime, as argued by Neely and Keefe (1989).

### Relatedness proportion

Numerous studies reported no influence of relatedness proportion on the semantic priming effect at short SOA (e.g., Grossi, 2006; Hutchison et al., 2001; Neely et al., 1989; Neely, 1977; Pecher et al., 2002; Perea and Rosa, 2002), which is consistent with the idea that semantic priming effects at short SOAs are due to automatic spreading activation or feature overlap as there is no time to form expectancy sets. Other studies have found that relatedness proportion does influence the semantic priming effect at short SOAs. For example, de Groot (1984) varied relatedness proportion (0.25, 0.50, 0.75, and 1.00) at different SOAs (240, 540, and 1,040 ms) and found an influence of relatedness proportion on the semantic priming effect when the SOA was 240 ms (the effect was bigger in the 0.75 condition than the 0.25 condition). de Wit and Kinoshita (2014, 2015a) also found that relatedness proportion modulated the semantic priming effect at SOAs considered too short for expectancy generation. de Wit and Kinoshita examined the effect of relatedness proportion on semantic priming using the lexical decision and semantic categorisation tasks (we address the influence of task type on the semantic priming effect in the subsequent section). The findings that relatedness proportion influences the semantic priming effect at short SOAs are consistent with the idea that semantic priming, at least in part, is driven by post-lexical meaning and integration accounts. When participants detect a relationship between a prime and target, they are biased to make a “word” response more quickly (Neely et al., 1989).

### Stimulus onset asynchrony (SOA)

Studies have also examined the semantic priming effect in associative and semantic prime-target pairs at varying SOAs. For example, Perea and Rosa (2002, Experiments 1 and 2) found that the semantic priming effect in the LDT was driven by association between prime and target at their shortest SOA (66 ms) but not at longer SOAs (83 ms and longer). Specifically, in their Experiment 1, the associative strength of semantically related prime target pairs influenced the magnitude of the priming effect only at the 66 ms SOA. In Experiment 2, Perea and Rosa used prime target pairs that were semantically related but not associatively related (at least based on association norms). Semantic priming was not found in the 66 ms SOA, but was found in the longer SOAs. The authors point to a hybrid model that combines automatic spreading activation and integration accounts of priming that may better explain such findings. The hybrid model proposes that at short SOAs (~60 ms), priming is brought on by automatic spreading activation, whereas at longer SOAs, integration of features plays a role. Importantly, the presence of priming that is automatic and driven by the associative relationship between prime and target is consistent with the conclusion of Hutchison (2003).

Bueno and Frenck-Mestre (2008, Experiments 5-7) likewise investigated the differences between semantically and associatively related primes in a masked priming paradigm using the LDT at various prime durations and backward masks yielding SOAs of 42 ms, 57 ms, 99 ms, and 256 ms. Their prime-target pairs were either associatively and semantically related, or just semantically related (i.e., feature overlap) but were not forward-associated according to word association norms. The authors found no semantic priming effect for either the associatively or semantically related pairs at the two shortest SOAs (Experiment 5), but did find a priming effect for both types of prime target pairs at the 99 ms SOA (Experiment 6), and at the 256 ms SOA (Experiment 7). Bueno and Frenck-Mestre, like Perea and Rosa (2002, Experiment 2), failed to find a semantic priming effect in the LDT for pairs that were only semantically but not associatively related when the SOA was short (42 ms and 57 ms SOAs for Bueno and Frenck-Mestre, and 66 ms SOA for Perea and Rosa). However, while Perea and Rosa found that the semantic priming effect was driven by prime-target association strength at their 66 ms SOA, Bueno and Frenck-Mestre did not find any priming based on association at their 42 ms and 57 ms SOAs. While Perea and Rosa used Spanish stimuli, Bueno and Frenck-Mestre used French stimuli. Perea and Rosa also did not use backward masks after presenting primes, meaning that in the 66 ms SOA condition, the prime was presented for 66 ms. Bueno and Frenck-Mestre used 14 ms backward masks in their two shortest SOA conditions, meaning that those primes were presented for 28 ms and 43 ms. Nonetheless, Bueno and Frenck-Mestre argue that their findings were consistent with those of Lucas (2000) and that automatic semantic priming does not depend on association strength.

Ball et al. (2024) examined the effect of SOA on semantic priming from primes that were newly learned words. The authors compared semantic priming effects from familiar word primes and novel word primes learned during the course of the study at varying SOAs (450 ms, 1,000 ms, 200 ms). In all cases, primes were presented for 200 ms, and the interstimulus intervals were varied to produce differing SOAs. The authors found that at the 450 ms and 200 ms SOAs, novel words did not prime their targets while familiar words did. At the 1,000 ms SOA, novel words did prime their targets, but familiar words did not. Ball et al. argue that because the semantic representations of novel words are not yet integrated into the semantic network (Davis and Gaskell, 2009), they engage in semantic processing through controlled and strategic semantic processes, which require greater SOAs (see also Bakker et al., 2015; Balass et al., 2010; Perfetti et al., 2005). Familiar words, in contrast, could semantically prime their targets automatically (i.e., at shorter SOAs).

Ball et al. (2024) link the strategic processing of primes (including retrospective integration) to longer SOAs. Specifically, Ball et al. argue that at a 1,000 ms SOA, the lack of semantic priming from familiar word primes was because of the low prime-target BAS. In contrast, the authors say that at the 1,000 ms SOA, retrospective integration may have contributed to the priming from the novel words as the BAS ratings between these primes and their targets were greater. These findings are potentially consistent with those of Hutchison et al. (2008), where greater BAS predicted a stronger semantic priming effect at a long SOA but not a short one. However, these findings contradict de Wit and Kinoshita (2015a, 2015b), where semantic priming was found in the LDT at a short enough SOA to preclude expectancy generation (we discuss de Wit and Kinoshita in more detail subsequently). De Wit and Kinoshita argue these effects are driven by retrospective matching because they were modulated by relatedness proportion and the priming effect was greater for slower trials (see also Taikh and Lupker, 2020). Based on Neely’s (1991) three process model of semantic priming, retrospective integration should be possible at shorter SOAs because the processing of the prime could continue during the processing of the target. Retrospective integration of novel word primes and their targets would thus allow priming at shorter SOAs, suggesting that the effects found by Ball et al. were driven by expectancy generation.

In summary, the literature suggests temporal boundary conditions for the mechanism driving semantic priming. The contribution of strategic prime processing to the semantic priming effect can only be completely ruled out when the prime is presented for less than 50 ms and thus cannot be identified. However, ruling out the influence of automatic processing of the prime is difficult because it is unclear how long it persists. When the prime can be identified, retrospective processing of the prime can also contribute to the semantic priming effect, and the use of this strategy is encouraged by a greater relatedness proportion, and stronger BAS. An SOA of approximately 300 ms is thought to be required for expectancy generation and is encouraged by a strong FAS. Variables that encourage faster processing of the prime and slower processing of the target (e.g., length and frequency) predict greater semantic priming effects.

The experimental task type influences the effects of modality and presentation variables. For example, tasks that require producing the targets (e.g., naming or typing the target words) seem to minimize the use of retrospective matching even if the SOA and BAS are sufficient to encourage retrospective matching. We address the effect of task-level determinants next.

## Task-level determinants

The semantic priming effect has been studied using numerous tasks (Balota et al., 2008; de Wit and Kinoshita, 2014; de Wit and Kinoshita, 2015a, 2015b), including semantic decision, naming, and relatedness judgment, although the lexical decision task (LDT) is the most common. In the LDT, participants decide whether a target letter string is a real word or a nonword. Most often, participants respond only to the target, not the prime. However, in the single-presentation LDT (e.g., Davies, 1998; Jones, 2010; McNamara and Altarriba, 1988), participants respond to both the prime and the subsequently presented target. Responses to the LDT are thought to be driven mainly by lexical level activity (e.g., Pexman et al., 2002), although post lexical strategies such as semantic matching may influence responses as well (e.g., Taikh and Lupker, 2020). However, several studies have shown limitations in using a lexical decision task. Some studies have shown the LDT to be a less reliable indicator of semantic processing than a semantic categorization task (Becker et al., 1997; Bueno and Frenck-Mestre, 2008; de Groot, 1990; Grainger, 1998; Williams, 1996).

### Single presentation-LDT

In the single-presentation LDT, participants respond to both the prime and the target sequentially. The single-presentation LDT is argued to be driven solely by automatic processes, thus excluding the influence of expectancy generation or retrospective matching because participants are thought to be less likely to notice relationships between the subsequently presented words. Perea and Rosa (2002, Experiments 3 and 4) tested the semantic priming effect with prime - target items that were associatively and semantically related, or just semantically related. When the interval between a response and the subsequently presented stimulus was short (200 ms in Experiment 3), Perea and Rosa found a significant semantic priming effect for prime - target pairs that were associatively and semantically related, and prime - target pairs that were just semantically related. When the response stimulus interval was long (1750 ms in Experiment 4), there was only a priming effect for the associatively related pairs. Perea and Rosa argued that priming driven by a semantic (but not an associative) relationship may persist for a short time (i.e., over 200 ms but under 1750 ms). In summary, it seems that associative information may drive priming automatically, via automatic spreading activation, or strategically, via expectancy generation and/or retrospective matching.

### Semantic categorization task

In semantic categorization tasks, participants make a judgement about the target word that relies on its meaning, for example indicating whether the target is an animal or an object. In contrast to the LDT, semantic categorization tasks require participants to attend to some semantic dimension of the targets. Semantic priming with this task has been found to be greater at long SOAs, where more time is allowed to process the relevant semantic information of the prime (e.g., Becker et al., 1997). As with the LDT, some researchers (e.g., Jared and Seidenberg, 1991) argue that semantic categorization may be contaminated by other processes, such as expectancy generation (Hutchison, 2003; Lucas, 2000). However, results from Bueno and Frenck-Mestre (2008) show that semantic categorization tasks are not necessarily strategy-dependent and may reflect automatic spreading activation. As noted, Bueno and Frenck-Mestre examine the semantic priming effect in semantically and associatively related pairs with various SOAs, in both the LDT (Experiments 5–7) and semantic categorization task (Experiments 1–4). The authors found a semantic priming effect in pairs that were semantically but not associatively related in the semantic categorization task at the 42 ms and 57 ms SOAs (Experiment 1), which contrasts with their Experiment 5, where no semantic priming effect was found for semantically but not associatively related pairs at these same SOAs when using the LDT. Using prime-target pairs that were associatively related but weakly semantically related, the authors found no priming at SOAs of 42 ms and 57 ms (Experiment 2), but did find priming at an SOA of 99 ms (Experiment 3) and an SOA of 256 ms (Experiment 4).

Considering their LDT and semantic categorization experiments, Bueno and Frenck-Mestre (2008) concluded that semantic priming increases when the task requires processing of semantic information. Specifically, priming effects occurred at shorter SOAs in the semantic categorization task than the LDT. Additionally, just having an associative relationship was not enough to produce priming at short SOAs, suggesting that associative information could drive priming via expectancy generation and/or retrospective matching. In contrast, a semantic relationship, which includes feature overlap, could be used quickly and automatically to drive semantic priming in the semantic categorization task. Thus, Bueno and Frenck-Mestre argue that the lexical decision task is not as sensitive to early semantic processing. Similarly, McRae and Boisvert (1998) argue that strategic processes in semantic categorization tasks can be reduced if researchers use broad categories.

In a series of experiments, de Wit and Kinoshita (2014, 2015a, 2015b) examined semantic priming effects in both the lexical decision task and the semantic categorization task. The authors likewise conclude that the mechanisms driving semantic priming are different across tasks. In the lexical decision task, semantic priming effects are due to retrospective semantic matching mechanisms, even when the SOA is too short to allow generation of expectancy sets. In semantic categorization tasks, semantic priming effects are due to automatic facilitation of the target by the prime, although differently from the way posited by spreading activation accounts. In a semantic categorization task (where participants make a judgement about the target word that relies on its meaning), the semantic prime facilitates its target via evidence accumulation and source confusion. Specifically, if the judgement is whether the target is an animal or not, participants accumulate evidence in the form of diagnostic features (e.g., “has four legs”) to make their decision (see also Norris, 2006). De Wit and Kinoshita propose that evidence accumulated from the prime gets combined with the evidence accumulated from the target (i.e., source confusion) when the two are presented in close temporal proximity. Thus, the strength of the featural relationship (i.e., how much of an overlap of features there is between the prime and target), not necessarily of the associative relationship, influences how strongly the prime can facilitate the target in a semantic categorization task. Retrospective matching should also be discouraged in the semantic categorization task (de Wit and Kinoshita, 2014). When the SOA between prime and target is short (eliminating the possibility of expectancy set formation) the evidence accumulated from the prime gets confused for evidence accumulated from the target, resulting in the related prime facilitating the target. Although, similar to the argument made by Jones (2010), retrospective matching could consist of attending to a semantic dimension of the target and prime, for example, deciding whether the prime and target belong to the “animal” category. Again, this would be retrospective matching, but not in a way that is consistent with the compound-cue model (McKoon and Ratcliff, 1992).

de Wit and Kinoshita examined the semantic priming effect in the semantic categorization task (de Wit and Kinoshita, 2014) and the lexical decision task (de Wit and Kinoshita, 2015a) as a function of relatedness proportion, with a 240 ms SOA (the prime was presented for 200 ms and there was a 40 ms interstimulus interval). An SOA of 240 ms should preclude the generation of expectancy sets, allowing for the prime to facilitate the target via automatic activation and/or a retrospective strategy. In both tasks, the authors found a greater semantic priming effect with a high relatedness proportion, ruling out an automatic spreading activation as posited by Collins and Loftus (1975) as the only mechanism driving semantic priming. De Wit and Kinoshita then examined the latency distributions of responses. Specifically, a semantic priming effect that is greater on slower trials would be consistent with a retrospective strategy being applied after lexical access to the target, since the prime would have more time to influence the response during slower trials. Of note, this is similar to the logic used by Thomas et al. (2012), who found a greater semantic priming effect for visually degraded targets (which took longer to process, thus allowing more time for the prime to influence the target), but only the prime and target pair had a backward association. de Wit and Kinoshita found that the semantic priming effect was indeed greater for slower trials in high relatedness proportion condition in the lexical decision task, but was similar across fast and slow trials for the semantic categorization task, suggesting that retrospective matching operated during the LDT but not semantic categorization.

Similarly, de Wit and Kinoshita (2015b) examined the semantic priming effect in the lexical decision and semantic categorization tasks when the primes were able to be consciously identified by participants (the prime was presented for 200 ms followed by a 40 ms blank) or not (prime presented for 50 ms and was immediately followed by the target). In both tasks, a semantic priming effect was found when the primes were visible. Making the prime unavailable for conscious identification eliminated the semantic priming effect in the lexical decision task but not in the semantic categorization task. Additionally, in the lexical decision task, the semantic priming effect was greater for trials with slower responses, while in the semantic categorization task, the size of the priming effect was similar between slower and faster trials. The authors argue their results are consistent with semantic priming being driven by a retrospective matching process in the lexical decision task and by pre-activation in the semantic categorization task. Specifically, eliminating the visibility of the prime (and thus eliminating the use of retrospective matching) eliminated the semantic priming effect in the lexical decision task but not the semantic categorization task. Although we should note that a 50 ms prime presentation may not entirely preclude prime identification (Hirshman and Durante, 1992).

The collective findings of de Wit and Kinoshita (2014, 2015a, 2015b) are not consistent with the previously discussed findings of Fernandino and Conant (2024), who found that the overlap in sensory and affective features was the primary driver of the automatic semantic priming effect in the LDT (the authors used a 200 ms SOA). It should be noted that while Fernandino and Conant only used the LDT, de Wit and Kinoshita directly compared the LDT and semantic categorization tasks.

The findings of Taikh and Lupker (2020) are also consistent with the semantic priming effect in the lexical decision task being driven by a retrospective process. Taikh and Lupker examined the concurrent effects of semantic primes (at long and short SOAs) and masked orthographic primes on target responses. Specifically, a semantic prime (*mutton*) and then an orthographic prime (*lkmb*) preceded a target (*LAMB*). While the mechanism(s) driving semantic priming are debated, the effect of the masked orthographic prime (which is usually not consciously identified) is thought to be automatic and pre-lexical. The effects of the semantic and orthographic primes did not interact, indicating that the two types of primes influenced separate processes according to Sternberg’s (1969) additive factors logic. Additionally, consistent with De Wit and Kinoshita (2014, 2015b), the semantic priming effect increased during slower trials. In contrast, the orthographic priming effect remained consistent between slow and fast trials.

### Naming

In naming tasks, participants read the target word aloud. Compared to the LDT, naming tasks may provide a purer measure of the influence of automatic spreading activation on semantic priming (Balota and Lorch, 1986; Seidenberg et al., 1984), as retrospective matching is less likely to be used for the demands of the naming task. Consistent with this, Hutchison et al. (2008) found that BAS was a stronger predictor of semantic priming in the lexical decision task than in naming at long SOA (see also Kahan et al., 1999). Overall, though, Hutchison et al. found reliable semantic priming effects in both the LDT and naming, although the effect was smaller in naming (Lupker, 1984; Neely, 1991).

Hutchison et al. (2008) found that FAS predicted the semantic priming effect in both the naming task and the LDT, while the BAS predicted the effect in only the LDT. The authors argued the retrospective matching mechanism contributes to semantic priming in the LDT, but not in naming. Heyman et al. (2016) also examined whether the mechanisms driving semantic priming in the LDT and naming are the same using a large semantic priming dataset. Heyman et al. used data from the Semantic Priming Project (Hutchison et al., 2013), a dataset of naming and LDT responses from 768 participants for 1,661 targets following related and unrelated semantic primes at both short (200 ms) and long (1,200 ms) SOAs. The authors reasoned that if the priming effects of the two tasks are driven by the same mechanisms, then item-level priming effects should be correlated. When controlling for prime and target properties (i.e., length, frequency, orthographic neighbourhood size, baseline response latencies in the English Lexicon Project), Heyman et al. found that item-level priming effects were correlated between the two tasks. However, correlations were stronger at the short SOA than the long SOA. When analyzing only items with a strong FAS and no BAS, these findings remained the same, suggesting that in both tasks, primes pre-activate their targets quickly and automatically via automatic spreading activation (Collins and Loftus, 1975) or the encoding of features (de Wit and Kinoshita, 2014, 2015a, 2015b).

### Typing

More recently, the typing task has been used to examine the processing and production of language (see Gagné et al., 2023a; Yamaguchi, 2019 for reviews). Typing a word requires multiple processes, including identifying it, accessing its semantic information, planning the sequence of keystrokes, and executing that sequence. Unlike the LDT and semantic categorization tasks, the typing production of a word unfolds over multiple keystrokes. Keystroke latencies can be recorded with precision, thus allowing researchers to examine how linguistic information influences the processes involved in typing. Specifically, the latency of the first keystroke is thought to reflect accessing the word, planning its motor output, and storing the sequence of letters in the graphemic output buffer. The latencies of the non-initial letters are thought to reflect the execution of the motor plan.

Modular theories of typing posit that typing processes are sequential, and are informationally isolated from one another. For example, the Context Retrieval and Updating Model (Logan, 2018; Logan and Crump, 2009, 2011) posits a word level, where a lexical item is selected, and a letter level, where the item is decomposed into letters and these letters are typed (see also Yamaguchi et al., 2017). Importantly, according to modular theories, linguistic information is not present during, and does not influence the execution of the motor plan. Information from semantic primes should thus influence the initial keystroke, but not the non-initial ones. Interactive theories (e.g., Gagné and Spalding, 2016) posit that typing processes are cascaded, and thus allow for linguistic information to also influence the execution of the motor plan (i.e., the initial and non-initial keystrokes).

Few studies have examined the influence of semantic priming on typing, and it is not clear whether semantic primes influence the execution of the motor plan in addition to recognizing the word and planning the keystrokes. For example, Chen et al. (2021) analyzed word typing times from TypeRacer, an online game where participants type short passages. The authors found that words that were more predictable based on their context sentence, were typed more quickly. However, Chen et al. did not differentiate between the latencies of the initial and non-initial keystrokes, and thus their findings do not indicate whether contextual predictability influenced keystroke execution. In the TypeRacer game, and other naturalistic copy-typing tasks where the entire passage is available, it is difficult to control the delay between a target word and its preceding contextual information, so the influences of strategic processing of semantic information are not clear.

Scaltritti et al., 2017 examined the influence of semantic primes (duration and SOA were 100 ms) on the typing of auditorily presented targets. Scaltritti et al. argue that semantic priming influences only the word recognition processes (i.e., only the first stage of the Context Retrieval and Updating model) and wanted to test whether information from semantic primes persisted during the execution of the motor plan during the typing of the target words. The authors found that related primes facilitated both the initial keystrokes and the inter-key intervals between the first and second keystrokes of auditorily presented targets, but not the average latencies of the non-initial keystrokes. Scaltritti et al. argue that their findings are potentially consistent with the cascaded typing processes as posited by interactive theories of typing, although the effect on the interkey intervals between the first and second keystrokes was small relative to the effect on the initial keystrokes. The short SOA used by Scaltritti et al. precluded the use of expectancy generation, leaving spreading activation and retrospective lexical matching to potentially influence the execution of the motor plan.

Mangat and Taikh (2024) examined the influence of having more time to process semantic primes on typing targets, using a short SOA (275 ms) and a long SOA (600 ms). In both cases, the prime was presented for 200 ms and the duration of the interstimulus interval mask varied (i.e., 75 ms or 400 ms), and targets were visually presented using the type-to-copy paradigm, where the target remains on the screen as it is being typed. Related primes facilitated the initiation of the target words, but there was no effect on any non-initial key latencies, which is consistent with modular theories of typing. Interestingly, both the initial and non-initial target keystrokes were typed faster at the long SOA. Mangat and Taikh suggest that the prime (whether related or unrelated) may have interfered with the production of the target, where the prime was automatically encoded and its letters stored in the graphemic output buffer interfering with planning and executing the keystrokes of the target. A longer interstimulus interval may have allowed participants to clear the graphemic output buffer prior to seeing the target word. Mangat and Taikh also noted that using a type-to-copy task may reduce reliance on linguistic information, including semantic information from the prime because participants may copy target letters.

Current studies thus do not provide strong support for semantic primes facilitating the execution of the motor plan while typing targets. However, findings from studies examining the typing of compound words in a semantic priming paradigm (e.g., Gagné and Spalding, 2014, 2016; Libben et al., 2021) suggest that semantic primes influence how the semantic information of the constituent parts of the compound is integrated during its typing. Numerous studies (e.g., Gagné et al., 2023b; Gagné and Spalding, 2014, 2016; Gow et al., 2024; Sahel et al., 2008; Will et al., 2006) have found a delay in typing between the words embedded in compounds and pseudo-compounds (e.g., a pause between the final *h* of *high* and the *l* of *light* when typing *highlight*). This boundary effect suggests that embedded words, rather than the entire word, are planning units of production, and that morphological information is active during the execution of the motor plan. Additionally, Taikh et al. found that, in typing compound words the linguistic properties of the second constituent influenced the typing of the first constituent. When the second constituent was more semantically transparent (i.e., the constituent maintained its standalone word meaning to a greater extent, such as *berry* in *blueberry*), the first constituent was typed more quickly. The findings of Taikh et al. suggest that keystroke execution is influenced by semantic information, where the meanings of the individual constituents are accessed and integrated during typing.

Several studies (Gagné and Spalding, 2014, 2016; Libben et al., 2021) have examined the typing of compound word targets in the semantic priming paradigm. These studies have confirmed the boundary effect, and the influence of the semantic information of the constituents on the production of the compound targets. In general, semantically opaque constituents (e.g., *wash* in *hogwash*) were typed more slowly because their representations might be suppressed in favor of the meaning of the whole compound. Importantly, the effect of the semantic prime was influenced by the semantic transparency of the compound’s constituents. For example, Gagné and Spalding (2016) found that facilitating access to the meaning of the first constituent (e.g., the related prime *chicken* facilitating *egg* in the compound *eggbeater*), slowed down typing of the second constituent. These studies suggest that the semantic information from the prime influences accessing and integrating the semantic information of the compound’s constituent morphemes during the typing of the compound.

In summary, task-level determinants can either encourage or preclude strategic processing. For example, the single presentation-LDT is thought to preclude strategic processing, while the typical LDT likely reflects the influence of both automatic and strategic processing (although researchers disagree on what drives semantic priming in the LDT). In general, binary response tasks such as the typical LDT and the semantic categorization likely encourage retrospective matching processes, such that stronger BAS predicts a greater semantic priming effect. Production tasks, such as naming and typing, may rely less on retrospective matching and thus may be better suited for examining the influences of prospective priming mechanisms (i.e., automatic spreading activation and expectancy generation).

## Toward an integrated account of semantic priming

Numerous integrated accounts (e.g., Neely et al., 1989; Perea and Rosa, 2002) allow for semantic priming to be driven by both automatic and strategic processes. For example, Perea and Rosa propose an integrated account of semantic priming, where the effect is driven by automatic spreading activation at short SOAs (60 ms or shorter), and by post-lexical integration at longer SOAs. To continue to develop an integrated understanding of semantic priming a key step is to separate the contributions of automatic and strategic processing of the prime, and similarly, the influences of multiple concurrent mechanisms that can drive the semantic priming effect. Our review of the literature on the semantic priming effect shows that numerous factors (prime-level lexical and semantic properties, prime-target relational factors, modality and presentation variables, and task-level determinants) jointly influence the processing of the prime and the semantic priming effect, and the effects of these factors are often conflated. The influence of these factors is generally greater when strategic processing is possible. In this section, we integrate findings about the influence of these factors on both the automatic and strategic processing of the prime, and examine the key challenges in developing an integrated understanding of semantic priming and suggest future directions.

### Considering automatic processing of the prime

Automatic processing of the prime occurs quickly and does not require attention or explicit awareness of the prime (Posner and Snyder, 1975), and has been conceptualized as automatic spreading activation of the prime pre-activating the lexical representation of the target (Collins and Loftus, 1975) or as the extraction of semantic features from the prime that facilitate the processing of the target (e.g., de Wit and Kinoshita, 2014, 2015a, 2015b). Studies that find the semantic priming effect when the prime is presented too briefly to be identified (typically less than 50 ms) are consistent with automatic prime processing (e.g., de Wit and Kinoshita, 2015b; Hirshman and Durante, 1992). Specifically, not identifying the primes does not allow for expectancy generation or post-lexical integration. However, ruling out the influence of post-lexical integration when the prime is visible is difficult. As Hirshman and Durante noted, even at 50 ms durations, primes can be identified and processed concurrently with the target, and they found evidence of post-lexical integration at those durations.

It is also not clear how long the influence of automatic spreading activation persists, although studies have attempted to estimate its duration. For example, Perea and Rosa found a semantic priming effect for semantically related pairs in the single presentation LDT at a short SOA (200 ms), but not a longer SOA (1750 ms). The authors suggested that automatic spreading activation, which they argued is what drives priming for semantically related pairs, persists for less than 1750 ms. However, the authors also note that the absence of a priming effect for these pairs at a long SOA could have been due to expectancy generation of items that are associatively (but not purely semantically) related to the prime. Items generated based on expectancy would thus interfere with the semantically (but not associatively) related pairs. Thus, it is difficult to rule out the influence of automatic spreading activation at an SOA of 1750 ms for purely semantically related pairs, and it is still unclear how long automatic spreading activation persists in prime-target pairs with a different relationship or in a different task.

Studies disagree on how the type of prime-target relationship influences automatic processing of the prime. Lucas (2000) concluded that there was no semantic priming effect driven only by automatic prime processing when the primes and targets were not semantically related but only associatively related (see Perea and Rosa, 2002 for a similar conclusion based on an experiment that also manipulated SOA). In contrast to Lucas, Hutchison (2003) argued that there was evidence for automatic priming based on association alone, and further argued that mediated priming is an example of this. Similarly, it is unclear how task demands influence automatic prime processing. de Wit and Kinoshita (2015b) found that at SOAs of 50 ms, when the prime supposedly could not be identified, there was a semantic priming effect in the semantic categorization task but not the LDT, arguing that feature extraction (i.e., distributed but not localist accounts of semantic memory) explain automatic priming effects. However, Hirshman and Durante (1992) found a semantic priming effect in the LDT using prime durations of 33 ms. Similarly, Bueno and Frenck-Mestre (2008) found a semantic priming effect for associatively related pairs in the LDT at SOAs of 42 ms, when primes were unlikely to be identified.

An important direction for future studies attempting to develop an integrated account of semantic priming is thus to delineate when automatic processing of the prime drives or contributes to the semantic priming effect. The difficulty ruling out retrospective matching, and to some extent, expectancy generation, and the fact that these two strategies can obscure the influence of automatic processing of the prime make this challenging. However, studies using the single presentation-LDT could examine the influence of numerous factors (e.g., semantic vs. automatic prime-target relationship, and prime-level lexical and semantic properties). Similarly, studies extending De Wit and Kinoshita (2015b) could examine the effect of primes that cannot be consciously identified (i.e., prime duration being 33 ms instead of 50 ms where primes can still be identified, see Hirshman and Durante, 1992) in numerous tasks. Additionally, Hutchison (2003) argues that mediated priming precludes the use of expectancy generation and retrospective matching, although other researchers (e.g., Jones, 2010; McKoon and Ratcliff, 1992) argue that mediated priming can be explained by strategic processing of the prime. Examining mediated priming using a single presentation-LDT, or in a standard LDT with short durations, would clarify the mechanism(s) driving mediated priming. Such studies would provide boundary conditions under which automatic processing of the prime influences the semantic priming effect.

### Considering strategic processing of the prime

With greater prime duration and SOA, strategic prime processing plays a greater role in driving the semantic priming effect. Studies examining the semantic priming effect suggest that numerous variables allow and encourage the use of expectancy generation and post-lexical integration strategies. Generating expectancy sets is possible if the prime is consciously identified, and SOA is long enough (estimated to be 300 ms or longer, see Neely, 1977). The use of expectancy generation should be more likely when the stimulus list has a high relatedness proportion, and when the prime-target relationship is consistent and obvious (e.g., prime and target are members of the same category). Retrospective processing of the prime should also be possible whenever the prime can be visually identified. In theory, retrospective processing does not require a minimum SOA because the prime is being processed concurrently with the target. However, retrospective processing of the prime makes a greater contribution to the semantic priming effect when processing of the target words is slowed down, either due to visual degradation of the target words (e.g., Balota et al., 2008; Borowsky and Besner, 1993; Stolz and Neely, 1995; Thomas et al., 2012), or when the prime is a higher frequency word (Hutchison et al., 2008). In general, Hutchison et al. argue that strategic processing of the prime has a greater impact on the target when the prime is processed more quickly, and when the target is processed more slowly (allowing the more time for the related prime to influence the processing of the target).

The demands of the experimental tasks also influence how the prime is processed and thus the contributions of expectancy generation and retrospective matching to the semantic priming effect. Naming or typing targets requires multiple sequential responses (i.e., articulating a sequence of sounds or executing a sequence of keystrokes), while semantic categorization and the LDT require a single response for the target. Balota and Lorch (1986; see also Seidenberg et al., 1984) argue that the demands of the naming task are much less likely to require retrospective matching (although expectancy generation is still possible). The findings of Hutchison et al. (2008), that BAS was a weaker predictor of priming in naming than the lexical decision task at a long SOA, are consistent with this notion. Using data from the Semantic Priming Project (Hutchison et al., 2013), Heyman et al. (2016); found that naming and LDT item-level priming effects were correlated, but this correlation was stronger at the short SOA. Together, the findings of Hutchison et al. (2008) and Heyman et al. (2015) suggest that naming reduces or eliminates retrospective processing of the prime. In the naming task, the semantic priming effect may be driven by automatic processing of the prime (i.e., automatic spreading activation or the extraction of features), or by expectancy generation, but the prime does not seem to be processed while the target is being produced. Studies examining the semantic priming effect in typing (e.g., Mangat and Taikh, 2024; Scaltritti et al., 2017) likewise suggest that the influence of the prime on the production of the target may be prospective, influencing the identification of the target and the planning of its production (i.e., the latency of the first keystroke), but not the execution of the motor plan (i.e., the non-initial keystrokes). However, the finding by Mangat and Taikh that initial and non-initial target keystrokes were typed more quickly when the SOA was long (following related and unrelated primes) suggests that in the typing task, the prime may be processed while the target is being typed. Specifically, the letters of the prime, related or unrelated, may have been encoded and stored in preparation for their output (although the prime was not typed in this task). Thus, processing the prime as a stimulus that may need to be typed may have interfered with the concurrent output of the target. We note that there have been relatively few studies examining the semantic priming paradigm in typing.

Developing an integrated account of semantic priming requires delineating when strategic processes, such as expectancy generation and retrospective matching, drive the semantic priming effect. One challenge to this is accounting for or eliminating the influence of automatic processing of the prime. Boundary conditions under which automatic processing contributes to semantic priming, as discussed above, would be informative. Similarly, it is difficult to rule out the influence of retrospective matching strategies to isolate the influence of expectancy generation. Using prime-target pairs that have a strong backward association but no forward association (Thomas et al., 2012) may allow for examining the influence of expectancy generation in various tasks. In addition to numerous contradicting results and arguments in the literature that were noted in the present review (e.g., Lucas, 2000 vs. Hutchison, 2003), another challenge to developing an integrated account of semantic priming is that many factors that influence semantic priming are studied in isolation (e.g., Hutchison et al., 2013). One solution to this problem is to use large datasets of semantic priming, which we discuss next. First, though, we note that some factors cannot be studied concurrently and their interactions cannot be observed. For example, it would not be possible to control the SOA between prime and target, or the time participants spend processing the prime, in studies where participants respond to both the prime and the target sequentially (e.g., single presentation-LDT).

### Using large datasets of semantic priming

The majority of studies examining the semantic priming effect utilize factorial designs, which makes examining the factors driving the effect more challenging (Hutchison et al., 2013). Specifically, in such studies, the researchers select stimuli based on a variable of interest (e.g., associative vs. categorial prime-target relations) and attempt to equate the stimuli on other variables that influence word recognition and semantic priming. Because many variables can potentially influence word recognition and the semantic priming effect, stimuli in semantic priming studies cannot be effectively equated on all the other relevant variables. For example, as Hutchison (2003) notes, the influence of prime-target co-occurrence is difficult to separate from the influence of semantic relatedness because prime-target pairs that are semantically related often occur in the same linguistic context. Hutchison et al. also note that factorial designs that use unusual items (e.g., very high vs. very low word frequency) may lead participants to change the way they process primes and targets, reducing the generalizability of these studies. Hutchison et al. (see also Buchanan et al., 2026) argue that large datasets of semantic priming data, such as their Semantic Priming Project, can overcome some of these limitations. The Semantic Priming Project includes naming and LDT responses to 1,661 target words from 768 participants. The target words follow both related and unrelated primes, allowing Hutchison et al. to estimate item-level semantic priming effects when averaged over participants. Hutchison et al. note that datasets that include many items would allow item characteristics to vary continuously.

We note, however, that in a dataset with many items and thus variation on many variables, the effect of a specific variable of interest (e.g., type of prime-target relationship) may be obscured by the other variables. Sibley et al. (2009) attempted to replicate effects of numerous linguistic variables on naming latencies that are reliably obtained in factorial experiments using large datasets. For example, the robust finding that words with irregular (vs. regular) pronunciations are named more slowly, but only when they are low frequency (e.g., Seidenberg, 1985; Taraban and McClelland, 1987), was generally not replicated using datasets from megastudies such as the English Lexicon Project (Balota et al., 2008). Sibley et al. also found only moderate item-level correlations in naming latencies between megastudy datasets. The authors argued that large databases may not be sensitive enough to detect subtle, but theoretically significant effects that can be detected using factorial studies. Factorial studies are used to deliberately create conditions where the variable of interest can be examined. Using large datasets is thus not always a suitable replacement for factorial experiments.

Another challenge of examining semantic priming effects is that they are generally not reliable. In addition to examining the reliability of item-level priming effects across tasks (LDT vs. naming) in the Semantic Priming Project dataset (Hutchison et al., 2013), Heyman et al. (2016) examined the reliability of the semantic priming effects within each task and SOA grouping (i.e., short and long SOA groups in the naming and LDT tasks). The authors split the participant sample of each of the four groupings of data in half and computed correlations of the item-level priming effects. The correlation coefficients were similar for the two tasks and were greater when the SOA was short (spearman-brown estimates were about 0.3) than when SOA was long (spearman-brown estimates were about 0.2). Heyman et al. (2016) note that estimates indicate that semantic priming effects have low reliability, and are similar to reliability estimates computed in other studies (e.g., Heyman et al., 2015). Similarly, Heyman et al. (2018) examined the reliability of item-level semantic priming effects across two different times. In their study, participants completed two sessions that were 4 weeks apart, where the stimuli used in each session were identical. There were 134 critical targets, where the prime-target relationships were diverse (e.g., synonyms, antonyms, subordinates…). The SOA was 200 ms (the prime was shown for 150 ms followed by a 50 ms ISI prior to the target), which was meant to discourage strategic processing of the primes. Heyman et al. (2018) found low item-level test–retest reliability (less than 0.30), which is consistent with the findings of Heyman et al. (2015) and other studies examining the reliability of the semantic priming effect (e.g., Heyman et al., 2016).

Heyman et al. (2016, 2018) note that increasing sample size would increase the reliability estimates of item-level semantic priming effects. Individual participants may vary on how strongly two concepts (i.e., the prime and its target) are related, and on strategies they may use when the prime duration and SOA allow for strategic processing. While individual differences in semantic priming effects are beyond the scope of the present paper (see Yap et al., 2017), averaging over participants to compute item-level effects introduces variability, and increasing the number of participants would reduce this variability. For example, Heyman et al. (2018) computed the split-half reliability of semantic priming effects from randomly selected participants from data from Tan and Yap (2016), who tested the masked priming effect and used a sample of 240 participants. Hutchison et al. (2013) obtained a split-half reliability of 0.70 from the complete Tan and Yap dataset, but only an estimate of 0.28 from a subset of the data that included 40 participants.

## Summary and concluding remarks

The semantic priming paradigm has been a key way in which researchers have tried to understand the representation and retrieval of knowledge. Given that the semantic priming effect in any particular situation can result from automatic and/or strategic processes, our aim is to, whenever possible, delineate boundary conditions between when semantic priming is driven by automatic vs. strategic processing of the prime. Consistent semantic priming effects are most likely when strategic influences are minimized. Most notably, greater semantic priming is predicted by primes that are processed more quickly (i.e., short and frequent words with few orthographic neighbors) when the SOA is short and automatic processes are more likely to drive the semantic priming effect. However, it is still not clear whether automatic processing drives the semantic priming effect only when the prime and target are semantically related (e.g., Lucas, 2000; Perea and Rosa, 2002) or if it is also possible when the prime and target are associatively related (Hutchison, 2003). It is also unclear whether automatic processing can drive semantic priming in the lexical decision task (e.g., Bueno and Frenck-Mestre, 2008; Hirshman and Durante, 1992), or not (e.g., de Wit and Kinoshita, 2015b).

In contrast, the influence of strategic processing is more likely to result in inconsistencies of the semantic priming effect because strategic processing is more likely to be influenced by the factors reviewed here. Examining how numerous factors jointly influence the semantic priming effect, especially when it is driven by strategic processing, is important for delineating boundary conditions under which automatic and strategic processing drive the semantic priming effect. For example, novel word primes are only effective in priming their targets when the SOA is long enough (i.e., 1,000 ms) to process them strategically (Ball et al., 2024), as they are not yet integrated into the semantic network. Similarly, Hutchison et al. (2008) found that at long SOA (1,250 ms), when strategic processing was more likely, priming was predicted by both FAS and BAS. However, at short SOA (250 ms), when strategic processing was less likely, Hutchison et al. found that priming was predicted only by FAS. Similarly, while relatedness proportion was found to influence semantic priming at shorter SOAs (e.g., de Groot, 1984; de Wit and Kinoshita, 2014; de Wit and Kinoshita, 2015a), its influence was stronger at longer SOAs, and numerous studies did not find the influence of relatedness proportion at short SOAs (e.g., Perea and Rosa, 2002). Collectively, such findings help to narrow down the boundary conditions under which semantic priming is driven by automatic or strategic processing of the prime.

However, as Hutchison et al. (2013) points out, many factors that influence semantic priming are studied in isolation. Continuing to examine the joint influence of numerous factors would contribute to delineating conditions where automatic and strategic prime processing contributes to the semantic priming effect. We note some outstanding questions about the automatic and strategic drivers of the semantic priming effect as a function of time available to process the prime in Table 1. For example, the field would benefit from studies that examine the joint influence of a prime’s semantic richness and relatedness proportion. Few studies have examined semantic priming in the typing task. As in the naming task, typing a target unfolds sequentially, rather than ending after a single keystroke as in the LDT, and this likely influences the strategic processing of the prime and how long this information persists. Again, we note that some factors, and their interactions, cannot be studied concurrently. For example, in a paradigm where the prime and the target are sequentially presented and the participant responds to both (such as the single presentation-LDT), it is impossible to control the SOA between prime and target and thus to examine the influence of SOA in such a task. While using large datasets of semantic priming may facilitate examining the joint effects of multiple variables on semantic priming (Hutchison et al., 2013), they may not be sensitive enough to detect subtle effects that are still theoretically important (Sibley et al., 2009). Specifically, factorial experiments are deliberately created to examine these variables by using specific types of stimuli, prime-target relationships, presentation variables, and task types. Thus factorial experiments and large datasets are complementary in continuing to examine the joint influences of multiple variables on the semantic priming effect.

**Table 1:** Outstanding questions about automatic and strategic influences on the semantic priming effect at different prime processing times.

Time to process prime	Driver(s) of semantic priming	Outstanding questions
Prime not visible (prime duration under 50 ms)	Automatic processing of prime	Do priming effects driven purely by automatic processing of the prime require a semantic prime - target relationship? Is automatic semantic priming driven by automatic spreading activation or feature overlap? What tasks allow automatic priming effects? This is especially pertinent to the standard LDT.
Prime is visible and SOA is short (under about 300 ms)	Both automatic and strategic processing of prime (although automatic processing is likely to be the dominant contributor)	Do retrospective integration mechanisms drive semantic priming in tasks that require producing the targets (e.g., typing or naming)? Do retrospective integration mechanisms drive semantic priming in mediated priming paradigms? How much time is required to process the meaning of the prime, thus allowing for prospective mechanisms to drive semantic priming? Are the contributions of retrospective matching and automatic prime processing to the semantic priming effect additive? Do lexical and semantic properties of the prime that facilitate its processing (e.g., greater frequency) facilitate both automatic and strategic drivers of semantic priming?
Prime is visible and SOA is medium or long (over 300 ms)	automatic and strategic processing of prime, although strategic processing becomes more dominant with longer SOA	Is expectancy generation possible in mediated priming paradigms? How long does the influence of automatic prime processing persist? What factors influence this period of time when automatic processing of the prime influences processing of the target? Are the contributions of expectancy generation, retrospective matching, and automatic prime processing to the semantic priming effect additive? What is the minimum SOA needed for novel word primes to produce a semantic priming effect?

In conclusion, both automatic and strategic processing of the prime can concurrently contribute to the semantic priming effect, where strategic processing can obscure the influence of automatic processing. Strategic processing of the prime is more likely than automatic processing to be influenced by a wide array of factors, such as prime-level lexical and semantic properties, prime-target relational factors, modality and presentation variables, and task-level determinants. To develop an integrated account of semantic priming, it is critical to delineate conditions when automatic or strategic processing is contributing to the semantic priming effect. Our review aimed to delineate these boundary conditions through highlighting the joint influence of various factors on the semantic priming effect when possible. We also note contradictory findings in the literature, and the limits of integrating multiple task types, modalities, and temporal parameters, and suggest future directions.
